# Development of PCR markers specific to *Dasypyrum villosum* genome based on transcriptome data and their application in breeding *Triticum aestivum*-*D. villosum#4* alien chromosome lines

**DOI:** 10.1186/s12864-019-5630-4

**Published:** 2019-04-15

**Authors:** Shijin Li, Jing Wang, Kunyang Wang, Jingnan Chen, Ke Wang, Lipu Du, Zhongfu Ni, Zhishan Lin, Xingguo Ye

**Affiliations:** 1 Institute of Crop Sciences, Chinese Academy of Agricultural Sciences, Beijing, 100081 China; 2 College of Agronomy and Biotechnology/State Key Laboratory of Agrobiotechnology, Key Laboratory of Crop Heterosis and Utilization (MOE), Key Laboratory of Crop Genetic Improvement (Beijing Municipality), China Agricultural University, Beijing, 100193 China; 3 School of Plant Science and Technology, Beijing University of Agriculture, Beijing, 102206 China; 4 National Key Facility of Crop Gene Resources and Genetic Improvement, Chinese Academy of Agricultural Sciences, Beijing, 100081 China

**Keywords:** Molecular markers, Transcriptome, Alien chromosome lines, *Dasypyrum villosum*

## Abstract

**Background:**

*Dasypyrum villosum* is an important wild species of wheat (*Triticum aestivum* L.) and harbors many desirable genes that can be used to improve various traits of wheat. Compared with other *D. villosum accessions, D. villosum#4* still remains less studied. In particular, chromosomes of *D. villosum#4* except 6V#4 have not been introduced into wheat by addition or substitution and translocation, which is an essential step to identify and apply the alien desired genes. RNA-seq technology can generate large amounts of transcriptome sequences and accelerate the development of chromosome-specific molecular markers and assisted selection of alien chromosome line.

**Results:**

We obtained the transcriptome of *D. villosum#4* via a high-throughput sequencing technique, and then developed 76 markers specific to each chromosome arm of *D. villosum#4* based on the bioinformatic analysis of the transcriptome data. The *D. villosum#4* sequences containing the specific DNA markers were expected to be involved in different genes, among which most had functions in metabolic processes. Consequently, we mapped these newly developed molecular markers to the homologous chromosome of barley and obtained the chromosome localization of these markers on barley genome. Then we analyzed the collinearity of these markers among *D. villosum*, wheat, and barley. In succession, we identified six types of *T. aestivum*-*D. villosum#4* alien chromosome lines which had one or more than one *D. villosum#4* chromosome in the cross and backcross BC_3_F_5_ populations between *T. durum*–*D. villosum#4* amphidiploid TH3 and wheat cv. Wan7107 by employing the selected specific markers, some of which were further confirmed to be translocation or addition lines by genomic in situ hybridization (GISH).

**Conclusion:**

Seventy-six PCR markers specific to chromosomes of *D. villosum#4* based on transcriptome data were developed in the current study and their collinearity among *D. villosum*, wheat, and barley were carried out. Six types of *Triticum aestivum*-*D. villosum#4* alien chromosome lines were identified by using 12 developed markers and some of which were further confirmed by GISH. These novel *T. aestivum*-*D. villosum#4* chromosome lines have great potential to be used for the introduction of desirable genes from *D. villosum#4* into wheat by chromosomal translocation to breed new wheat varieties.

**Electronic supplementary material:**

The online version of this article (10.1186/s12864-019-5630-4) contains supplementary material, which is available to authorized users.

## Background

*Dasypyrum villosum* (2n = 14, VV) is a cross-pollinating annual grass in the tribe Triticeae, which is native to the north-eastern part of the Mediterranean region (from southern France to the Caspian Sea), south-western Asia, Russia and the Caucasus areas. It is a vigorous ruderal plant that can grow in harsh and moisture-stressed environments [1]. *D. villosum* is resistant to many major wheat diseases including stripe rust (*Puccinia striiformis*), leaf rust (*P. recondita*), stem rust (*P. graminis*), powdery mildew (PM) caused by *Blumeria graminis* (*Bgt*), eyespot (*Pseudocercosporella herpotrichoides*) and wheat spindle streak mosaic virus transmitted by *Polymyxa graminis* [1–5]. In addition, *D. villosum* is tolerant to cold, salt, and drought stresses, has a high tillering rate, produces large amounts of seed, and has high protein content [1]. Therefore, *D. villosum* has great potential as a genetic source for improving cultivated wheat with more desirable traits. As of now, more than 300 *D. villosum* accessions have been collected [6], but only the five accessions designated as *D. villosum#1*, *D. villosum#2*, *D. villosum#3*, *D. villosum#4,* and *D. villosum#5* have been introduced into wheat backgrounds [6–11].

The successful transfer of an arm or fragment of a chromosome from *D. villosum* into wheat background is an important step towards exploiting the desirable genes in *D. villosum*. To breed wheat with the aforementioned desirable traits of *D. villosum* and of the *T. durum*-*D. villosum* amphidiploid, some *T. aestivum*-*D. villosum* addition, substitution, and translocation lines have been developed using different *D. villosum* accession-derived materials as donors [6–11]. For example, six disomic addition lines (DA1V#1, DA2V#1, and DA4V#1-DA7V#1, except DA3V#1), seven monomer addition lines, excluding 3V#1, and two substitution lines, 1V#1/1A and 1V#1/1B, were created by using *D. villosum#1* [12, 13]. For *D. villosum#2*, a complete set of disomic addition lines (DA1V#2 to DA7V#2), substitution lines 2V#2(?), 3V#2(3D), 4V#2(4D), 5V#2(5D), and 6V#2(6A), and several translocation lines including T1V#2S·1BL, T1V#2L·1DL, T1V#2S·1DS, T1V#2S-6BS·6BL, T2V#2S·2DL, T4V#2S·4DL, T5V#2S·5AL, T5V#2S·5DL, T6V#2S·6AL, and T6V#2L·6AS have been obtained [4, 10, 14–19]. By using *D. villosum#3*, a complete set of disomic addition lines (DA1V#3 to DA7V#3), and some translocation lines including T1V#3S·1DL, T1V#3L·1DS, T2V#3L·2BL, T2V#3S·4BS, T2V#3S·4AL, T3V#3S·3DL, T3V#3L·3DS, T4V#3S·4DL, T4V#3L·4DS, T5V#3S·5DL, T6V#3S·6AL, T6V#3L·6AS, T7V#3S·7DL, and T7V#3L·7DS were developed [7, 9, 20, 21]. As for *D. villosum#4*, one substitution line 6V#4(6D) and three T6V#4S·6DL translocation lines (Pm97033, Pm97034, and Pm97035) were developed [11, 22]. For *D. villosum#5*, only one disomic addition line, DA2V#5, one disomic substitution line, 2V#5L(2D), and one translocation line, T2V#5L·2BS have been obtained [6]. However, a complete set of wheat alien addition lines, substitution lines or translocation lines involving each chromosome of *D. villosum#4* have not yet been created. By using a complete set of addition, substitution or translocation lines, the effect of an individual *D. villosum#4* chromosome on wheat growth and development can be identified and some useful genes in *D. villosum#4* can be located for wheat improvement [23].

*D. villosum* has many excellent genes, and the exact locations of some agronomic important genes on chromosomes are becoming clear (Table 1). Using the *T. aestivum*-*D. villosum#2* translocation line T6V#2S·6AL, carrying *Pm21*, and *T. aestivum*-*D. villosum#4* translocation line T6V#4S·6DL, carrying *PmV*, as PM resistance sources, some commercial wheat varieties including Neimai11, Neimai836, Yangmai18, Yangmai21, Yangmai97G59, Zhenmai9, Shimai14, Lantian27, Jinghe9123 and Yangmai22 have been developed and released in different areas [24]. However, most studies on *D. villosum* have been focused on *D. villosum#1*, *D. villosum#2*, and *D. villosum#3*, and investigations on *D. villosum#4* and *D. villosum#5* have been relatively scarce.

**Table 1:** Some agronomic important genes located on chromosomes of *D. villosum*

Characters	Loci	Chromosomes	References
HMW-glutenins polymeric	*Glu-V1*	1V #1	[62]
Sulfur-poor ω-type monomeric	*Gli-V1*	1V #1	[30, 63]
Sulfur-rich LMW polymeric prolamins	*Glu-V3*	1V #1	[63]
α-Prolamins	*Gli-V3*	4V#1L	[30]
Sulfur-rich α-prolamins	*Gli-V2*	6V#1S	[30]
Water-soluble endosperm protein	*Wsp-1*	7V#1	[64]
Increasing wheat grain protein content	*NAM-V1*	6V#2S	[65]
Powdery mildew (*Erysiphe graminis*)	*Pm21*	6V#2S	[14, 66, 67]
	*PmV*	6V#4S	[11, 24, 56]
	*Stpk-V*	6V#2S	[68]
	*Pm55*	5V#2S	[19]
	*CMPG1–V*	6V#2L	[69]
	*PDI-V*	5V#2	[70]
	*LecRK-V*	5V#2	[71]
	*Pm62*	2VL#5	[6]
Eyespot (*Pseudocercosporella herpotrichoides*)	*Pch Dv*	4V#1L	[72]
Wheat spindle streak mosaic virus	*Wss1*	4V#2S	[73]
Stem rust (*Puccinia graminis*)	*Sr52*	6V#3L	[7]
Cereal cyst nematode	*CreV*	6V#2L	[74]
Dark amber seed color	*R-V3*	3VL	[2]
Seeds softness genes	*Dina-D1a/Dinb-D1a*	5V#2S	[75]
Photoperiod response gene	*Ppd-V1*	2V#2S	[18]
Controlling bristles on the glume ridges	*Bgr-V1*	2V#2S	[18]

Although SSR [25, 26], ISSR [27], EST-PCR [28], and STS [29] markers distributed on chromosomes 1V to 7V, especially on chromosome 6VS [24, 30–38] in *D. villosum* have been reported, most of these markers are developed based on the *D. villosum#2* accession. Wide genetic diversity is known to exist among different original accessions such as *D. villosum#2* and *D. villosum#4*. Previous studies have shown that there were structural divergences between *D. villosum#2* and *D. villosum#4* based on cytogenetic observations, 6VS-specific molecular markers, and differing reactions to wheat curl mite [24, 31, 39, 40]. The marker MBH1 can tag both *Pm21* and *PmV*, from 6V#2S and 6V#4S, respectively, showing different sized amplification fragments [24]. In order to efficiently obtain addition, substitution, and translocation lines involved in each chromosome of *T. aestivum*-*D. villosum#4*, it is necessary to develop convenient molecular markers specific to each chromosome arm of *D. villosum#4*.

High-throughput RNA-seq technology can generate large amounts of transcriptome sequences and accelerate the development of chromosome-specific molecular markers. Recently, RNA-seq has been frequently used to develop molecular markers specific to chromosomes of wild relatives of cultivated wheat. For example, *Thinopyrum intermedia* genome-specific EST-SSR markers, *Agropyron cristatum* chromosome 6P-specific EST markers, *Aegilops longissima* chromosome-arm specific PCR markers and *D. villosum#4* chromosome 6V#4S-specific PCR markers were developed using transcriptome data [31, 41–43]. Thereby, RNA-seq is a potential strategy to develop molecular markers specific to different chromosome arms of wild species of wheat.

In this study, we obtained the transcriptome data of *D. villosum#4* by using RNA-seq and generated unigene sequences by transcriptome assembly after removing the transcripts from wheat by reference genome matching. Then, molecular primers specific to *D. villosum#4* chromosomes 1V to 7V were designed based on the unigene sequences. Furthermore, six types of *T. aestivum*-*D. villosum#4* candidate alien chromosome lines derived from the cross of *T. durum*–*D. villosum#4* amphidiploid TH3 and wheat cv. Wan7107 followed by a backcross were identified by using a portion of the developed markers and genomic in situ hybridization (GISH). The results obtained in this study will be useful to breed addition, substitution and translocation lines of *T. aestivum*–*D. villosum#4* and subsequent wheat varieties by marker assisted selection (MAS).

## Results

### Analysis of *D. villosum#4* transcriptome data

**Fig. 1:**
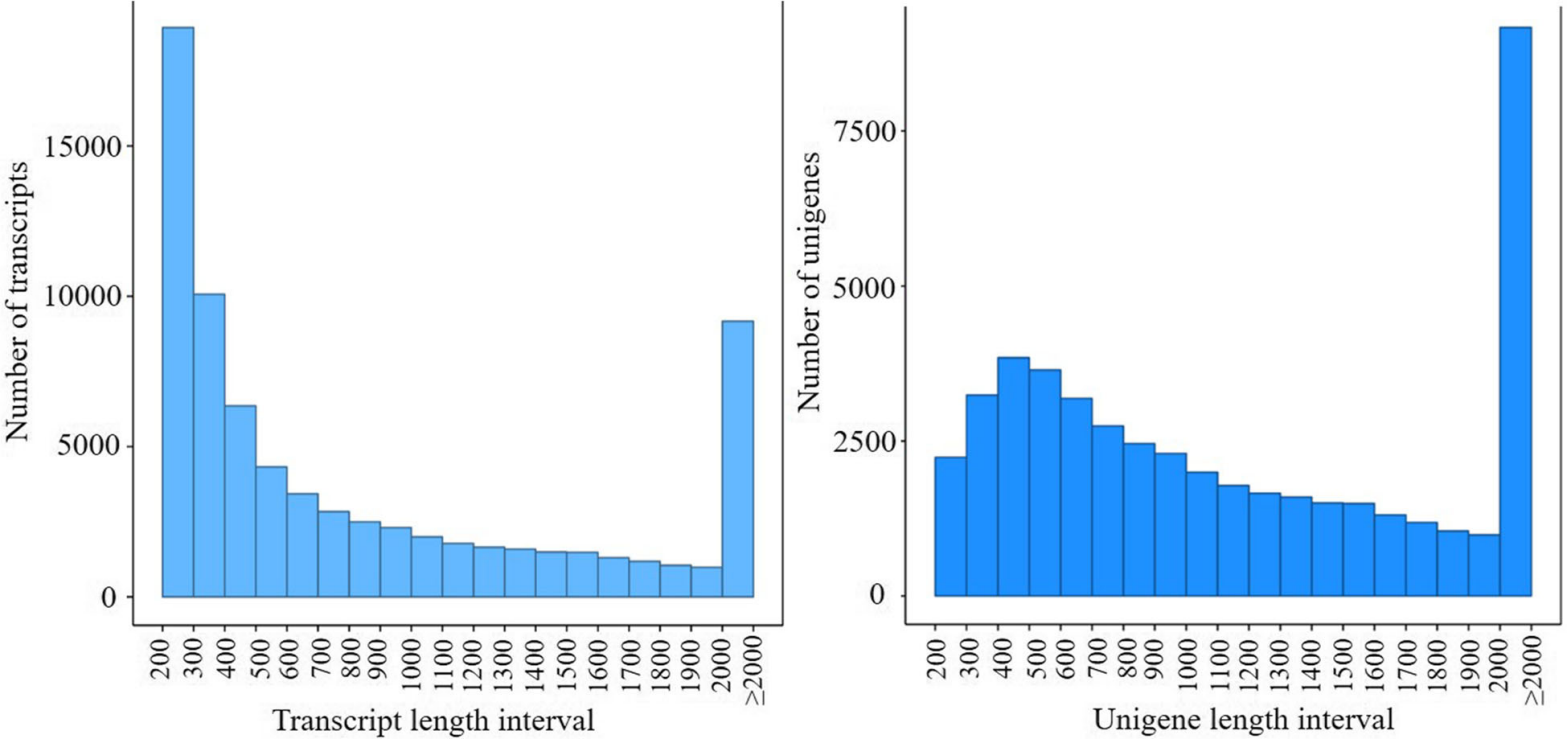
Distribution of transcripts (left panel) and spliced unigenes (right panel) of various lengths from *D. villosum#4* RNA-seq data. The *x*-axis is the sequence length intervals in base pairs, and the *y*-axis is the number of spliced transcripts or unigenes found within each length interval

**Fig. 2:**
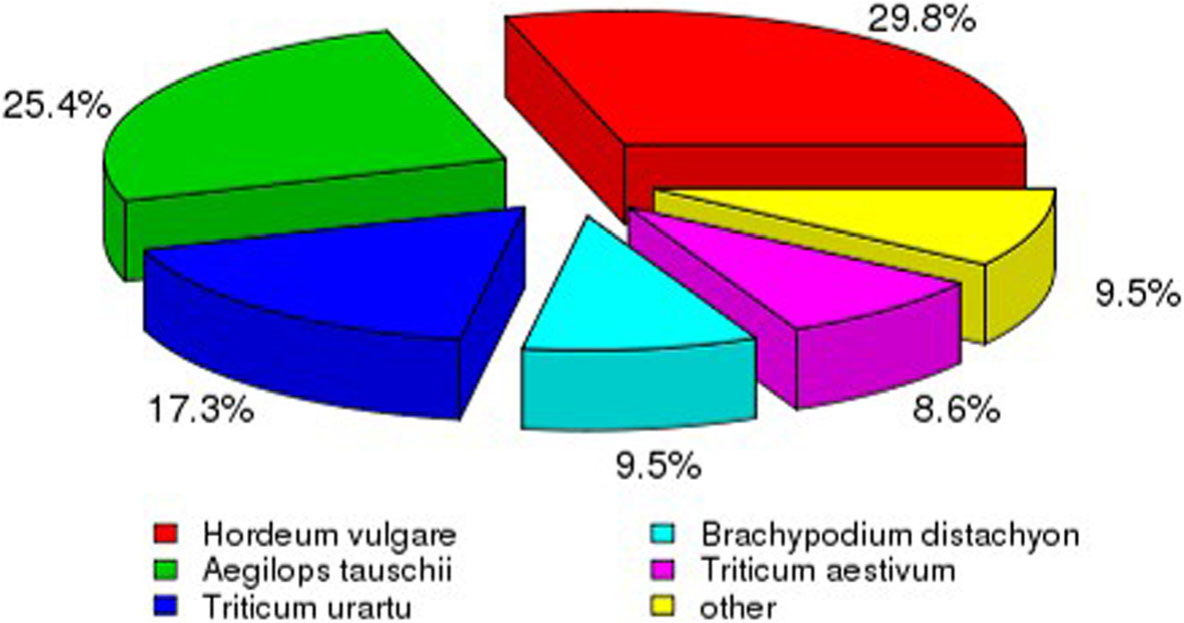
Plant species classification of the transcriptome unigene matching to a non-redundant (Nr) library. 29.8% (red), 25.4% (green), 17.3% (navy blue), 9.5% (light blue), 8.6% (purple), and 9.5 (yellow) of the unigene sequences are homologous to those in *Hordeum vulgare*, *Aegilops tauschii*, *T. urartu*, *Brachypodium distachyon*, *T. aestivum*, and other/unknown, respectively

We carried out transcriptome sequencing of *D. villosum#4* accession No. 1026, and obtained 7.14 G of clean bases and 47,619,528 clean reads in total. The rate of base mismatch was 0.02% (Table 2). Then, we spliced the clean reads and acquired a total of 74,517 transcript sequences and 47,382 unigene sequences (Fig. 1). The number of unigenes and transcripts sequences that had greater than 301 bp were 45,128 and 55,449, respectively. These sequences can be readily used for marker development. Seven databases were used for gene functional annotation of the 47,382 unigenes obtained in this study. The percentages of those unigenes annotated in Nr, Nt, Pfam, KOG/COG, Swiss-Prot, KEGG, and GO databases were 74.24, 4.31, 51.53, 28.5, 50.27, 25.19, and 52.29%, respectively. When comparing the unigene sequences to the non-redundant (Nr) library, we found that 29.8, 25.4, 17.3, 9.5, 8.6, and 9.5% of the unigene sequences were homologous to those in *Hordeum vulgare*, *Aegilops tauschii*, *T. urartu*, *Brachypodium distachyon*, *T. aestivum*, and other/unknown, respectively (Fig. 2).

**Table 2:** Quality analysis of *D. villosum#4* RNA-seq data

Sample	Raw reads	Clean reads	Clean bases	Error (%)	Q20 (%)	Q30 (%)	GC (%)
DV#4	47,619,528	47,619,528	7.14G	0.02	97.35	93.04	57.28

### Primary selection of *D. villosum#4* genome-specific markers

**Fig. 3:**
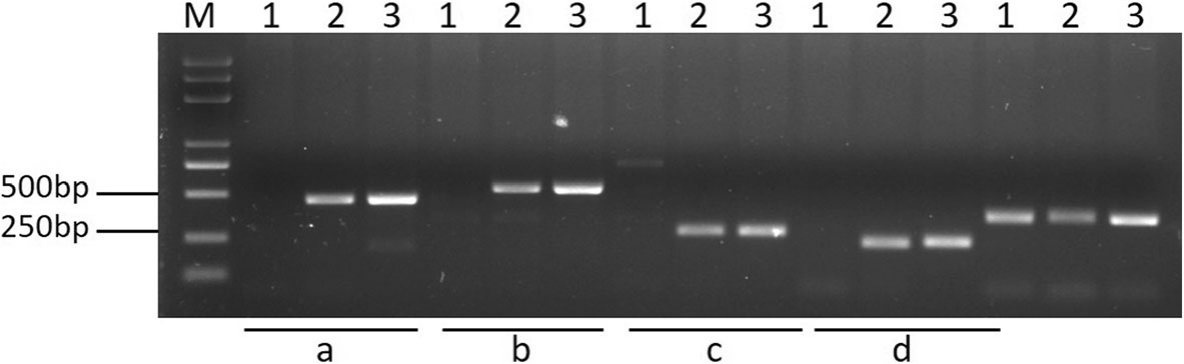
Amplification of candidate primers specific to *D. villosum#4* targeted chromosomes designed by *D. villosum#4* RNA-seq unigenes. M: marker 5000; 1: CS; 2: DA5V#3; 3: *D. villosum#4* accession No. 1026. a: amplified by marker 5V-13; b: amplified by marker 5V-40F3R3; c: amplified by marker 5V-14; d: amplified by marker 5V-15

Those unigenes having the highest similarity to the sequences on the chromosomes of wheat groups 1 to 7 were considered candidate markers specific to *D. villosum#4* chromosomes 1V to 7V. Primers were designed based on these unigene sequences, and further used to amplify the DNA of CS, one corresponding candidate line of the seven addition lines from DA1V#3 to DA7V#3, and *D. villosum#4* accession No. 1026 for the screening of *D. villosum#4* chromosome-specific amplicons. Figure 3 is an example of candidate markers specific to *D. villosum#4* chromosome 5V. The amplification pattern of the candidate primers a, b, c, and d were verified at the next step (Fig. 3).

### Confirmation of markers specific to *D. villosum#4* chromosomes 1V to 7V

**Fig. 4:**
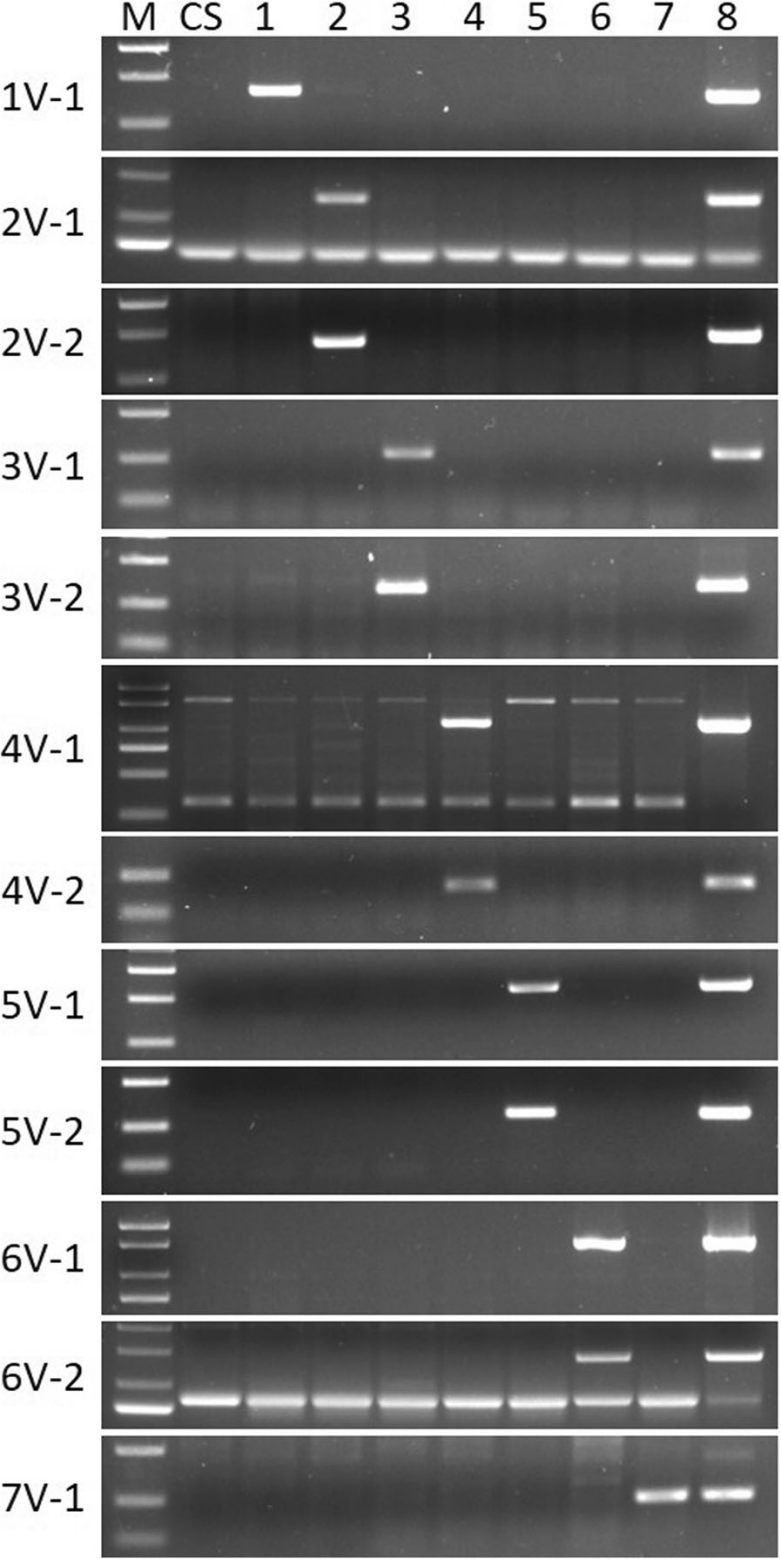
Amplification patterns of 12 different representative chromosome-specific primers in CS, 7 *T. aestivum*-*D. villosum#3* addition lines, and *D. villosum#4* accession No. 1026. M: marker 2000+; CS: Chinese Spring; 1: DA1V#3; 2: DA2V#3; 3: DA3V#3; 4: DA4V#3; 5: DA5V#3; 6: DA6V#3; 7: DA7V#3; 8: *D. villosum#4* accession No.1026

To develop specific molecular markers to each chromosome of *D. villosum#4*, seven *T. aestivum*-*D. villosum#3* addition lines (DA1V#3, DA2V#3, DA3V#3, DA4V#3, DA5V#3, DA6V#3, and DA7V#3), No. 1026 and CS were amplified with the selected candidate primers, in which CS was used as the negative control, and No. 1026 was used as the positive control. Results showed that some primers amplified a unique band in *D. villosum#4* and their corresponding addition line (one of the seven addition lines from DA1V#3 to DA7V#3) containing the targeted *D. villosum#3* chromosome, and did not amplify the same band in CS (Fig. 4); some primers did not amplify any unique bands in CS and the seven addition lines (DA1V#3 to DA7V#3), only amplifying unique bands in *D. villosum#4* No. 1026 (Additional file 1). There are abundant polymorphisms between the *D. villosum#4* accession used for RNA-seq and the *D. villosum#3* accession used as the alien chromosome donor of the seven addition lines in this study. However, some primers amplified unique alien band in an unexpected addition line, other than in the expected corresponding addition line. Finally, 76 *D. villosum#4* chromosome-specific primers, most of which can specifically amplify bands in addition lines of DA1V#3 to DA7V#3, were obtained, including 1 in 1V#4, 9 in 2V#4, 27 in 3V#4, 10 in 4V#4, 15 in 5V#4, 13 in 6V#4, and 1 in 7V#4 (Additional file 2). These specific molecular markers can potentially be used to trace different chromosome arms of *D. villosum#4* carrying useful genes for wheat breeding.

### Comparative analysis of the developed markers specific to *D. villosum#4* genome with wheat and barley genome

As described above, we developed the markers specific to *D. villosum#4* chromosomes 1V to 7V based on those unigenes which had the highest similarity to those sequences on the chromosomes of wheat groups 1 to 7. However, the *D. villosum#4* transcriptome data (Fig. 2) showed that the *D. villosum#4* unigene sequences had the most homology with *Hordeum vulgare*. To clarify the collinearity of the 76 newly developed specific PCR markers with the corresponding sequences among *D. villosum*, wheat, and barley. The chromosome localization of these markers on the barley was carried out by BLASTN (Additional file 2). It is illustrated that most corresponding sequences of the molecular markers specific to 1V to 6V have the same chromosome localization in wheat and barley, including one marker specific to 1V, 7 of 9 markers specific to 2V, 23 of 27 markers specific to 3V, 4 of 10 markers specific to 4V, 14 of 15 markers specific to 5V, 6 of 13 markers specific to 6V (Additional file 2).

### Functional annotation of *D. villosum#4* genes harboring the specific molecular markers

The 76 specific molecular markers were developed based on *D. villosum#4* transcriptome sequences, and thus were expected to correspond to *D. villosum#4* or *D. villosum*-associated genes. The sequences containing the 76 specific molecular markers were annotated in detail using the Nr, Nt, Pfam, KOG/COG, Swiss-prot, KEGG, and GO databases (Table 3). The annotations revealed that these markers were involved in different genes; most genes had functions in metabolic processes, and 22 genes had no annotation. For example, markers 6V-10, 6V-11, and 6V-12 were annotated to phyB activation tagged suppressor 1 (*BAS1*), which is a gene that regulates brassinosteroid levels and light responsiveness in *Arabidopsis* [44]. However, it is unknown whether a similar functioning gene may exist in *D. villosum#4*. Additionally, marker 5V-9 was annotated to E3 ubiquitin ligase which can catalyze the protein.

**Table 3:** Functional annotation of the 76 *D. villosum#4* genes identified in this study

Markers	Annotation
1V-1, 2V-9, 3V-2, 3V-3, 3V-4, 3V-14, 3V-15, 3V-16, 3V-17, 3V-18, 3V-19, 3V-24, 4V-5, 4V-7, 4V-8, 5V-1, 5V-4, 5V-5, 5V-7, 5V-12, 5V-13, 5V-14	None
2V-1	Plastid and cyanobacterial ribosomal protein (PSRP-3/Ycf65)
2V-2	CAAX protease self-immunity
2V-3, 2V-4, 2V-5, 2V-6, 2V-7, 2V-8,	Expansin-like B1
3V-1	Stomatin-like protein 2
3V-5, 3V-12, 3V-23	*Triticum aestivum* chromosome 3B, genomic scaffold
3V-6	biosynthetic process
3V-7	Probable serine/threonine-protein kinase
3V-8, 3V-9, 3V-10	G-type lectin S-receptor-like serine/threonine-protein kinase
3V-11	Dihydropyrimidinase
3V-13	Rhodanese-like domain-containing protein 4
3V-20, 3V-21, 3V-22	3-methyl-2-oxobutanoate hydroxymethyltransferase 1
3V-25	intracellular protein transport
3V-26	protein dimerization activity
3V-27	structural constituent of ribosome
4V-1	Serine/threonine-protein phosphatase PP1
4V-2	Photosystem I P700 chlorophyll
4V-3	Putative disease resistance protein RGA3
4V-4	signal transduction//regulation of transcription, DNA-templated//cell proliferation//growth
4V-6	Phosphoinositide phosphatase SAC8
4V-9, 4V-10	hypothetical protein F775_00277
5V-2	RNA-binding protein 1
5V-3	Probable tuliposide A-converting enzyme b6
5V-6	microtubule-based movement//microtubule-based process
5V-8	putative glycerophosphoryl diester phosphodiesterase 1
5V-9	E3 ubiquitin ligase
5V-10	nitrate assimilation
5V-11	ATPase ASNA1 homolog
5V-15	Glutathione S-transferase zeta class
6V-1, 6V-2, 6V-3	Calcineurin subunit B
6V-4	Detoxification
6V-5	Probable LRR receptor-like serine/threonine-protein kinase
6V-6, 6V-7, 6V-8	Transcription factor
6V-9	oxidoreductase, Chitin binding
6V-10, 6V-11, 6V-12	PHYB activation tagged suppressor 1
6V-13	Ulp1 protease family
7V-1	Immortalisation up-regulated protein//B-box zinc finger

### Application of *D. villosum#4*-specific markers in breeding new *T. aestivum-D. villosum#4* alien chromosome lines

**Fig. 5:**
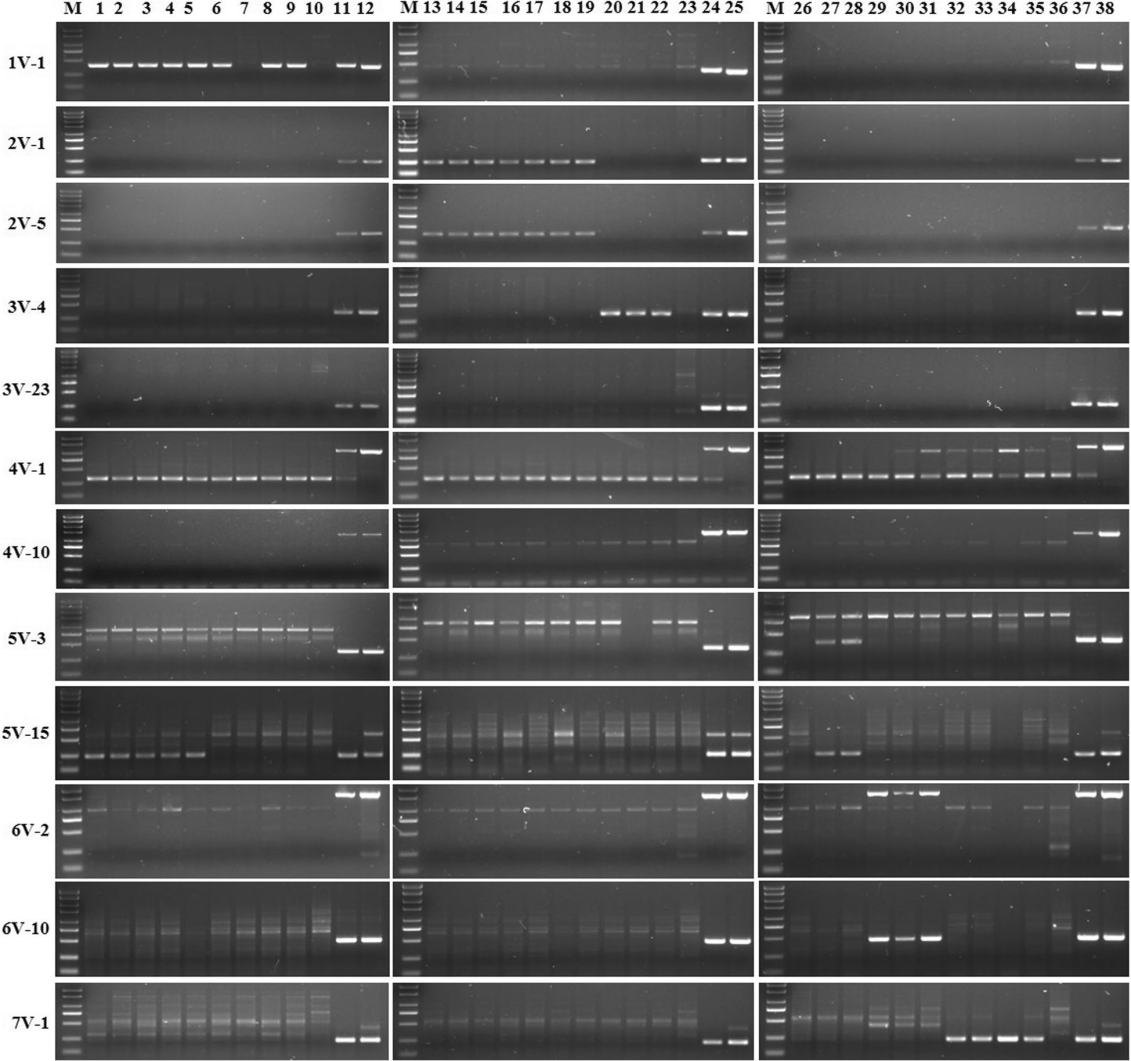
Amplification patterns of 12 primers specific to *D. villosum#4* 1V to 7V. M: marker; 1–5: plants 201–1-1, 201–1-2, 201–1-3, 201–1-4, and 201–1-5, respectively; 6–9: plants 201–2-1, 201–2-3, 201–2-4, 201–2-5, respectively; 10, 23, and 36: W7107; 11, 24, and 37: DA1V#3, DA2V#3, DA3V#3, DA4V#3, DA5V#3, DA6V#3, and DA7V#3, respectively, from top to bottom in each column; 12, 25, and 38: *D. villosum#4* accession No. 1026; 13–19: plants 203–1-1, 203–1-2, 203–1-3, 203–1-4, 203–1-5, 203–1-7, and 203–1-9, respectively; 20–22: plants 203–2-1, 203–2-2, and 203–2-3, respectively; 27–28: plants 204–2-2 and 204–2-3; 29–31: plants 204–3-1, 204–3-2, and 204–3-3, respectively; 32–35: plants 204–4-1, 204–4-2, 204–4-3, and 204–4-4, respectively

In order to develop new *T. aestivum*–*D. villosum#4* alien chromosome lines which contain *D. villosum#4* chromosomes 1V to 7V, a BC_3_F_5_ population consisting of 409 plants from the cross and backcross between the *T. durum*–*D. villosum#4* amphidiploid TH3 and the wheat line Wan7107 was screened using 12 developed PCR markers specific to *D. villosum#4* chromosomes 1V to 7V. Six types of *T. aestivum*-*D. villosum#4* alien chromosome lines were obtained except 4V (Table 4). The specific primers 1V-1, 2V-1, 2V-5, 3V-4, 3V-23, 5V-3, 5V-15, 6V-2, 6V-10 and 7V-1 amplified different specific bands in *D. villosum#4* No. 1026, the corresponding addition lines (DA1V#3, DA2V#3, DA3V#3, DA5V#3, DA6V#3, and DA7V#3), and the *T. aestivum*-*D. villosum#4* candidate alien chromosome lines; however, they did not amplify any band in wheat line Wan7107 (Fig. 5).

**Table 4:** Identification of candidate new *T*. *aestivum*–*D. villosum#4* alien chromosome lines using specific markers in BC_3_F_5_ generation from the cross of TH3 and Wan7107

Plants ID	Specific markers											
	1V	2V-1	2V-5	3V-4	3V-20	4V-1	4V-10	5V-3	5V-15	6V-2	6V-10	7V
201-1-1	+								+			
201-1-2	+								+			
201-1-3	+								+			
201-1-4	+								+			
201-1-5	+								+			
201-2-1	+											
201-2-2	+											
201-2-4	+											
201-2-5	+											
201-2-6	+											
202-1-1		+	+									
202-1-2		+	+									
202-2-1		+	+									
202-3-1		+	+									
202-3-2		+	+									
202-3-3		+	+									
202-3-4		+	+									
202-3-6		+	+									
202-3-7		+	+									
202-3-8		+	+									
202-3-9		+	+									
203-1-1		+	+									
203-1-2		+	+									
203-1-3		+	+									
203-1-4		+	+									
203-1-5		+	+									
203-1-7		+	+									
203-1-9		+	+									
203-2-1				+								
203-2-2				+								
203-2-3				+								
203-2-4			+									
203-2-5				+								
204-1-1	+	+	+	+	+	+	+	+	+	+	+	+
204-2-2								+	+			
204-2-3								+	+			
204-3-1										+	+	
204-3-2										+	+	
204-3-3										+	+	
204-4-1												+
204-4-2												+
204-4-3												+
204-4-4												+

**Fig. 6:**
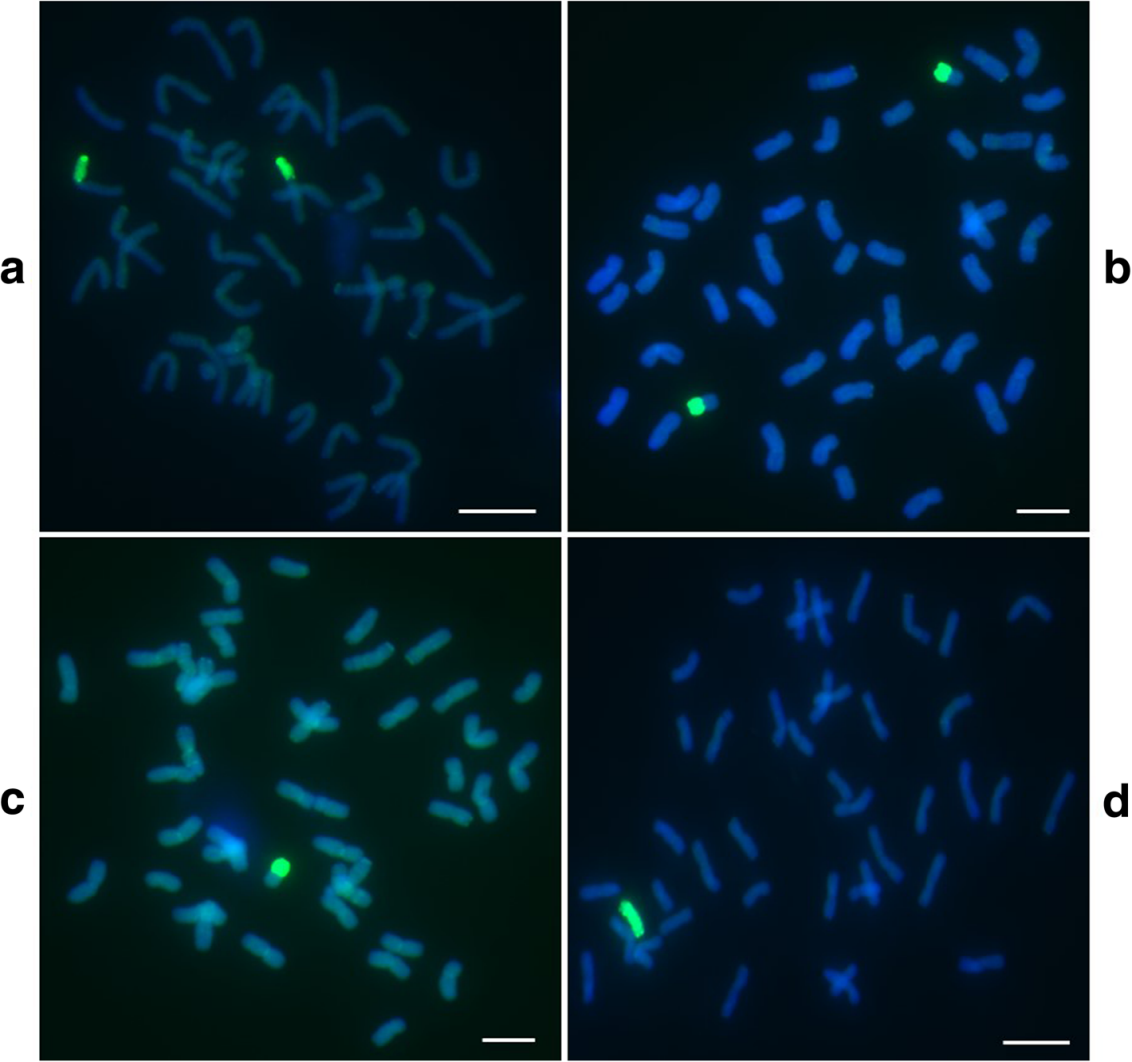
Identification of the candidate *T. aestivum*–*D. villosum#4* alien chromosome lines by GISH. Two translocated chromosomes between wheat and *D. villosum#4* were clearly observed in plant 201–1-3 (**a**). A pair of translocated chromosomes between wheat and *D. villosum#4* were clearly observed in plant 201–2-1 (**b**). One translocated chromosome between wheat and *D. villosum#4* was clearly observed in plant 201–2-4 (**c**). Forty-three chromosomes including one *D. villosum#4* chromosome were clearly observed in plant 204–2-3 (**d**). The scale bar in each photo is 10 μm in length

Due to the fact that markers 1V-1 and 5V-15 are located on *D. villosum#4* chromosomes 1V and 5V, respectively, and markers 1V-1 and 5V-15 amplified specific bands for plants 201–1-1, 201–1-2, 201–1-3, 201–1-4, and 201–1-5, these plants most likely contain *D. villosum#4* chromosomes 1V and 5V (Additional file 2). Then, the plant 201–1-3 were assayed by GISH using the genomic DNA of *D. villosum#4* as a probe. As a result, two combined chromosomes between wheat and *D. villosum#4* were clearly observed in this plant, indicating that plant 201–1-3 is a translocation line involved in *D. villosum#4* 1V and/or 5V (Fig. 6a).

**Fig. 7:**
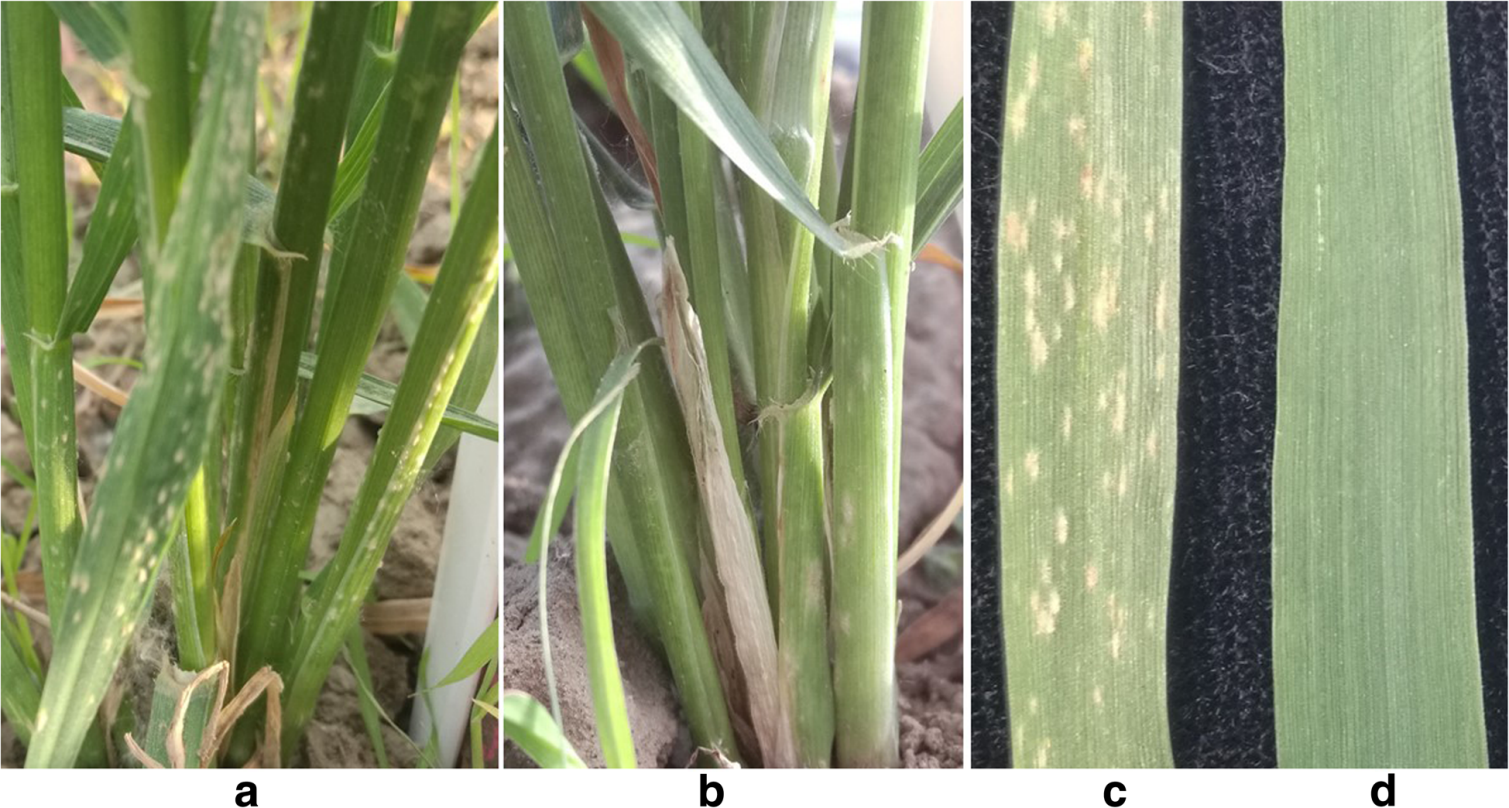
Responses of adult plants 201–2-1 (**a** and **c** only containing *D. villosum#4* chromosome 1VS) and 201–1-3 (**b** and **d** only containing *D. villosum#4* chromosomes 1VS and 5VS) to *Bgt* at the booting stage. Plant 201–1-3 showed high PM resistance while plant 201–2-1 displayed serious susceptibility to the disease, which was consistent with the results detected by molecular markers and GISH

Marker 1V-1 amplified specific bands from plants 201–2-1, 201–2-4, and 201–2-5, indicating that these plants most likely contain *D. villosum#4* chromosome 1V. Plants 201–2-1 and 201–2-4 were further assayed by GISH, and results indicated that two and one combined chromosomes between wheat and *D. villosum#4* were clearly observed in the two plants, respectively, demonstrating that plants 201–2-1 and 201–2-4 are translocation lines of wheat chromosomes and *D. villosum#4* 1VL or 1VS (Fig. 6b and c). The marker and GISH detection results of these plants are consistent with their responses to exposure to *Bgt*. Plants 201–1-1, 201–1-2, 201–1-3, 201–1-4, and 201–1-5 showed high resistance to *Bgt* (Fig. 7). Based on our marker and GISH detection results, these plants most likely contain *D. villosum#4* chromosome 5VL or 5VS. According to a previous study, a PM resistance gene *Pm55* was identified to be located on *D. villosum#2* chromosome 5VS [19]. Therefore, we inferred that there might be a homologous gene of *Pm55* on *D. villosum#4* chromosome 5VS. So the plants 201–1-1, 201–1-2, 201–1-3, 201–1-4, and 201–1-5 most likely contain *D. villosum#4* chromosome 5VS. In contrast, plants 201–2-1, 201–2-3, 201–2-4, and 201–2-5 were highly susceptible to *Bgt* because they only carry chromosome 1V (Fig. 7).

Markers 5V-3 and 5V-15 amplified specific bands from plants 204–2-2 and 204–2-3, indicating that these plants most likely contain *D. villosum#4* chromosome 5V. The GISH detection in plant 204–2-3 showed forty-three chromosomes including one *D. villosum#4* chromosome which were clearly observed in the plant, indicating that plant 204–2-3 is a monosomic addition line of wheat and *D. villosum#4* 5V (Fig. 6d).

## Discussion

This study was conducted based on transcriptome data from *D. villosum#4*. Currently, RNA-seq has been widely used in plant science for finding new transcripts, understanding gene expression patterns, excavating single-nucleotide polymorphisms (SNPs), exploring RNA alternative splicing and gene structural variation due to its high sequencing depth [45–50]. Particularly, RNA-seq has been extensively used to fully reveal the global gene expression profile at a specific point in time and a specific location (e.g. 0, 24, 48 h after inoculation of leaves with *Bgt*). For non-model plants with limited genomic sequence information, application of RNA-seq can be used to discover gene coding regions despite the small number of repetitive elements and high GC contents compared to whole genomes, which makes the assembly of transcriptome data relatively easy [51]. RNA-seq is thus a potential technique for developing molecular markers in some plant taxa, especially those with limited existing genomic sequences.

Compared with general molecular markers, the PCR markers developed from transcription data have particular advantages in plant genetics and breeding [52]. Firstly, transcription data contain a large amount of transcript information, and the PCR marker based on transcription data is related to a definite trait and the corresponding transcript of this marker may be directly associated with the gene controlling this trait [53]. Secondly, the transcript sequences are highly conserved in their homologous genes, so the PCR markers developed based on transcription data are versatile in function and would be useful for modifying linkage maps or comparative maps among closely related species [54, 55].

The transfer of the foreign chromosomes into wheat through alien chromosome lines to confer desirable new traits also brings undesirable foreign genes. Nevertheless, wheat alien chromosome lines are very important foundational materials in chromosome engineering and wheat breeding programs. At present, research regarding the utilization of *D. villosum* focuses primarily on germplasm innovation. Wheat distant breeding using *D. villosum* will allow introduction of some desired genes into the general wheat background. This process is likely to be more successful when wheat*-D. villosum* alien chromosome lines are used as a bridge. Currently, wheat-*D. villosum* amphidiploid, addition, substitution, and translocation lines have been bred already. In order to efficiently identify *D. villosum* chromatin in the wheat background, including its targeted chromosomes and arms or fragments, it is necessary to develop a large number of markers specific to each chromosome arm or region of *D. villosum*. Current research on *D. villosum*, including marker development, is mostly focused on the accession *D. villosum#2*. Although some preliminary investigations on *D. villosum#4* have been performed [11, 22, 31, 37, 39, 56], this genetic resource has not yet been fully exploited and utilized. Developing markers specific to the *D. villosum#4* genome will accelerate the application of the potential genes in *D. villosum#4* and further expand the genetic background of wheat.

To develop molecular markers specific to *D. villosum#4* using RNA-seq without knowing the chromosome locations of all the unigene sequences obtained by analyzing the transcriptome data, we initially submitted the unigene sequences to the URGI BLAST database https://urgi.versailles.inra.fr/blast/ to screen for unigene sequences which have high similarity to the sequences on the chromosomes of wheat groups 1 to 7. It was thus assumed that these unigene sequences were located on the chromosomes 1V to 7V of *D. villosum#4*. This might be a convenient approach to develop markers specific to the chromosomes of *D. villosum#4* in the wheat background. Following this strategy, we located the *D. villosum#4* unigenes to wheat genome, and then designed the specific molecular markers based on the differences between these unigenes in wheat and *D. villosum#4* genomes.

Finally, we developed 76 PCR markers specific to the chromosome arms of *D. villosum#4*. Among them, enough markers specific to the chromosomes 2V, 3V, 4V, 5V, and 6V were obtained. The results revealed by transcriptome analysis indicated that these unigene sequences had the most homology with the corresponding sequences from *Hordeum vulgare* (Fig. 2). Further, we located the locations of the developed molecular markers on the barley H genome (Additional file 2), which will provide a reference for the localization of these molecular markers specific to *D. villosum#4* in the wild relative species of common wheat. Based on the locations of these markers on the wheat groups 1–7 as compared with their locations on the barley H genome, we found that the locations of 55 markers specific to *D. villosum#4* chromosomes were consistent with their chromosomal locations in wheat and barley (Additional file 2). These results suggest that there are some affinities among the three species. Eight markers including 2V-1, 2V-2, 4V-1, 4V-2, 4V-3, 6V-10, 6V-11, and 6V-12 display different chromosomal location in *D. villosum#4,* wheat, and barley, although these markers share the same chromosomal locations in wheat and barley (Additional file 2). Five markers of 4V-9, 4V-10, 5V-12, 6V-7, and 6V-9 have the same chromosomal location in *D. villosum#4* and wheat, but different from that in barley. Four markers 3V-2, 3V-3, 3V-4, and 4V-4 have the same chromosomal location in *D. villosum#4* and barley, but different from that in wheat (Additional file 2). Four markers 3V-1, 6V-4, 6V-13, and 7V-1 show different chromosomal location among the three plant species (Additional file 2). A similar phenomenon was also found in a previous report [29]. According to our results, the high homologous sequence of marker 2V-1 is located on chromosomes 5DS in wheat and 5H in barley, while it is not specifically amplified in the 5V addition line and it is specifically amplified in the 2V addition line on the contrary; the high homologous sequences of markers 3V-2, 3V-3, and 3V-4 are located on chromosome 5DL in wheat and 3H in barley, while they are not specifically amplified in the 5V addition line and they are specifically amplified in the 3V addition line; the high homologous sequences of markers 4V-1, 4V-2 and 4V-3 are located on chromosomes 7DS, 7AS and 5DL in wheat and 7H, 5H in barley, while they are not specifically amplified in the 7V, 5V addition line and they are specifically amplified in the 4V addition line (Additional file 2). This phenomenon suggested that there were some synteny among chromosomes 2V in *D. villosum#4*, wheat homoeologous groups 2, and 5, and barley 2H and 5H, between chromosomes 3V in *D. villosum#4* and wheat homoeologous groups 3 and 5, and among chromosomes 4V in *D. villosum#4*, wheat homoeologous groups 4, 5 and 7, and barley 4H, 5H and 7H. Previously, it was reported that 59 4A ESTs could detect the loci on 5BL and 5DL, 72 4A ESTs could detect the loci on 7AS and 7DS, which confirmed the translocations involving chromosomes 4AL, 5BL, and 7AS in wheat [57]. This finding could explain part results of altered chromosomal location abovementioned. Taking together, the results aforementioned indicate that the collinearity of the developed markers on the chromosomal regions in *D. villosum#4,* wheat, and barley is interrupted. These structural differences reflect that the three plant species have undergone different evolutionary processes.

Update, more than 300 *D. villosum* accessions have been collected from different native habitats, and only a few (*D. villosum* #1 to *D. villosum*#5) have been used to develop wheat-*D. villosum* chromosome addition, substitution, and translocation lines [6–21]. There is some genetic divergence among different *D. villosum* accessions [24, 31, 39]. In this study, we were only able to develop one marker each for 1V and 7V. This phenomenon may be caused by the relationship between the chromosomes of *D. villosum#4* and wheat, and the polymorphism between different sources of *D. villosum*. While developing the markers specific to 1V and 7V of *D. villosum#4*, we found that most primers (86.67% in 1V and 70.59% in 7V) could not amplify any unique band in DA1V#3-DA7V#3, but that they could amplify bands in *D. villosum#4*. This indicated that there might be polymorphisms in the 1V and 7V between the accessions of *D. villosum#4* and *D. villosum#3*. The specific reasons for this phenomenon need to be further studied. We will also try to map the correspondence *D. villosum#4* unigenes to H genome for the development 1V- and 7V-specific markers in the near future.

## Conclusions

*Dasypyrum villosum* is an important wild species of wheat and contain a lot of novel genes that can be used to improve agronomic traits of wheat. Most studies on *D. villosum* have focused on *D. villosum#1*, *D. villosum#2*, and *D. villosum*#3, whereas the number of investigations on *D. villosum#4* is relatively less. We obtained the transcriptome data of *D. villosum#4* by using RNA-seq. Then, 76 molecular markers specific to *D. villosum#4* chromosomes 1V to 7V were developed based on transcriptome data. Furthermore, six types of *T. aestivum*-*D. villosum#4* alien chromosome lines were identified by using 12 developed markers and some of which were further confirmed by GISH. These novel *T. aestivum*-*D. villosum#4* chromosome lines have great potential to be used for the introduction of desirable genes from *D. villosum#4* into wheat by chromosomal translocation to breed new wheat varieties.

## Methods

### Plant materials and cultivation

*D. villosum*#4 accession No. 1026 and wheat lines Chinese Spring (CS) and Wan7107, were maintained in our laboratory. *T. aestivum*-*D. villosum#3* addition lines DA1V#3, DA2V#3, DA3V#3, DA4V#3, DA5V#3, DA6V#3, and DA7V#3 in CS background were kindly provided by Prof. Wenxuan Liu of Henan Agricultural University. A backcross BC_3_F_5_ population derived from the cross of *T. durum*–*D. villosum#4* amphidiploid TH3 and wheat line Wan7107 was made by our laboratory. *T. durum*–*D. villosum#4* amphidiploid TH3 (2n = 42, AABBVV) is derived from the cross between *T. durum* cv. Mexicali75 (2n = 28, AABB) and the *D.villosum#4* accession No.1026 that originated from the former USSR. Wheat lines CS and Wan7107 and *D. villosum#4* No. 1026, DA1V#3, DA2V#3, DA3V#3, DA4V#3, DA5V#3, DA6V#3, and DA7V#3 were grown in a greenhouse. The backcross population of plants from the cross of TH3 and Wan7107 were grown in the field.

### RNA-seq and transcriptome assembly

Fresh leaves of *D. villosum#4* accession No. 1026 were collected at seedling stage and RNA was extracted using TRIZOL (Thermo Fisher Scientific Inc., Shanghai, China). RNA quality, purity, integrity, and concentration were tested by the methods described in our previous publications [31, 43]. The RNA-seq was carried out at Beijing Novogene Company, Beijing, China (http://www.novogene.com/). Libraries for sequencing were generated using NEBNext® Ultra™ RNA Library Prep Kit for Illumina® (NEB, USA). Then, the libraries were sequenced and paired-end reads were generated on the Illumina Hiseq platform. Clean data were obtained by removing the low quality reads after the raw data was refined through in-house Perl scripts. The high quality clean data were used for further analyses. Transcriptome assembly was conducted using the Trinity software with the ‘--min_kmer_cov’ parameter for a mismatch rate set to 2 by default, and all other parameters were also set to the default [58].

### Gene functional annotation

Gene function annotation was performed using Nr, Nt, Pfam, KOG/COG, Swiss-Prot, KO, and GO databases.

### Development of markers specific to the genome of *D. villosum#4* based on RNA-seq data

The strategy for screening candidate markers specific to the genome of *D. villosum#4* was previously described by Li et al. [31]. Primers were designed using Primer Premier 6.0 (http://www.premierbiosoft.com/primerdesign/) based on those candidate unigene sequences which have the highest similarity to the sequences on the chromosomes of wheat groups 1 to 7.

### DNA extraction and PCR amplification

Leaves were sampled from two-month-old seedlings of wheat lines CS and Wan7107, *D. villosum* accession#4 No. 1026, wheat-*D. villosum#3* addition lines DA1V#3, DA2V#3, DA3V#3, DA4V#3, DA5V#3, DA6V#3, and DA7V#3, and the backcross population of TH3 and Wan7107, and stored at − 80 °C. Then, the genomic DNA for developing specific makers and alien chromosome lines was isolated from all samples using a NuClean Plant Genomic DNA Kit (CW Bio Inc., China). And the genomic DNA used for probe in GISH was extracted from *D. villosum*#4 using the CTAB method. Finally, the DNA was dissolved in 50 μL ddH_2_O.

The PCR reactions of 15 μL volume included 100 ng of template DNA, 0.3 μL primer (10 μmol·L^− 1^), and 7.5 μL 2 × Taq MasterMix (containing Mg^2+^ and dNTP, CW Bio Inc., China). Amplification reaction was performed in a MiniAmp Plus thermal cycler (USA) with a PCR program including 95 °C for 5 min, 35 cycles of 20 s at 95 °C, 30 s at 60 °C, 1 min at 72 °C, and 8 min for 72 °C. Amplification products were run on a 2% agarose gel, and observed under UV light after Genecolour II (Gene-Bio, Beijing, China) staining.

### Chromosomes localization of *D. villosum#4*-specific markers on the barley H genome

The corresponding sequences of the 76 developed molecular markers specific to *D. villosum#4* chromosomes were submitted onto website http://plants.ensembl.org/Multi/Tools/Blast?db=core to obtain chromosomal localization information through BLASTN searching.

### GISH identification of new alien chromosome lines between *T. aestivum* and *D. villosum#4*

Wheat seeds were soaked in water containing 0.7% H_2_O_2_ for 24 h and put on three layers of moist filter paper in petri dishes. When the root tips grow to 2–3 cm in length, they were cut off and put into a 0.5 mL centrifuge tube (a hole was drilled on the lid of the tube before using). Then, the root tips were treated with nitrous oxide for 2 h in a special container and fixed in 90% acetic acid for 5 min. They were rinsed twice with sterile water. The apical milky parts of the root tips were excised and digested with cellulase and pectinase for 55 min. Cell spreading preparations at mitosis were made as previously described [43, 59, 60]. *D. villosum#4* genomic DNA was labeled with fuorescein-12-dUTP as a probe using the nick translation method according to the manufacturer’s instructions. GISH was performed as previously description [43, 59–61]. Hybridization signals were captured under an Olympus BX-51 fluorescence microscope equipped with a charge-coupled device system.

## Additional files

Additional file 1: Amplification patterns of six 1V and 7V candidate chromosome-specific primers in Chinese (CS), seven *T. aestivum*-*D. villosum#3* addition lines, and *D. villosum#4* accession No. 1026. M: marker 2000+; CS: Chinese Spring; 1: DA1V#3; 2: DA2V#3; 3: DA3V#3; 4: DA4V#3; 5: DA5V#3; 6: DA6V#3; 7: DA7V#3; 8: *D. villosum#4* accession No.1026. (DOCX 95 kb)

Additional file 2: Localization of the 76 developed PCR markers in wheat, barley, and *D. villosum#4*, and their primer sequences and amplified sizes. 1: Chromosomal localization of the corresponding homologous sequences of the developed markers in wheat; 2: Chromosomal localization of the corresponding homologous sequences of the developed markers in barley; 3: Chromosomal localization of the corresponding sequences of the developed markers in *D. villosum#4*. (DOCX 24 kb)

## Abbreviations

*Bgt*: *Blumeria graminis* f. sp. *tritici*
COG: Clusters of orthologous groups of proteins
CS: Chinese Spring
DA: Disomic addition
EST: Expressed sequence tag
GISH: Genomic *in situ* hybridization
GO: Gene ontology
HMW: High molecular weight
ISSR: Inter-simple sequence repeats
KEGG: Kyoto encyclopedia of genes and genomes
KOG: Eukaryotic ortholog groups
LMW: Low molecular weight
MAS: Marker assisted selection
Nr: NCBI non-redundant protein sequences
Nt: NCBI nucleotide sequences
Pfam: Protein family
PM: Powdery mildew
SNPs: Single-nucleotide polymorphisms
SSR: Simple sequence repeats
STS: Sequence-tagged site
