# Supplementary material for: Development of PCR markers specific to Dasypyrum villosum genome based on transcriptome data and their application in breeding Triticum aestivum-D. villosum#4 alien chromosome lines
Source: BMC Genomics. 2019 Apr 15;20:289. doi: 10.1186/s12864-019-5630-4 (PMC6466811; doi:10.1186/s12864-019-5630-4)
Supplement: Supplementary file 1 — Amplification patterns of six 1V and 7V candidate chromosome-specific primers in Chinese (CS), seven T. aestivum-D. villosum#3 addition lines, and D. villosum#4 accession No. 1026. M: marker 2000+; CS: Chinese Spring; 1: DA1V#3; 2: DA2V#3; 3: DA3V#3; 4: DA4V#3; 5: DA5V#3; 6: DA6V#3; 7: DA7V#3; 8: D. villosum#4 accession No.1026. (DOCX 95 kb) [file 12864_2019_5630_MOESM1_ESM.docx]

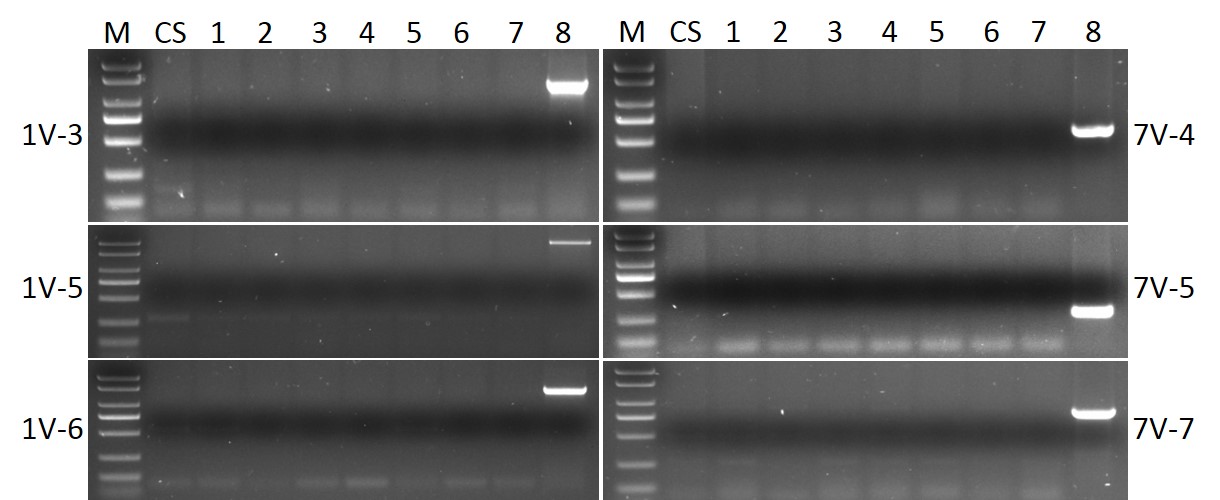


**Additional file 1:** Amplification patterns of six 1V and 7V candidate chromosome-specific primers in Chinese (CS), seven *T. aestivum*-*D. villosum#3* addition lines, and *D. villosum#4* accession No. 1026. M: marker 2000+; CS: Chinese Spring; 1: DA1V#3; 2: DA2V#3; 3: DA3V#3; 4: DA4V#3; 5: DA5V#3; 6: DA6V#3; 7: DA7V#3; 8: *D. villosum#4* accession No.1026.
