# Supplementary material for: Development of PCR markers specific to Dasypyrum villosum genome based on transcriptome data and their application in breeding Triticum aestivum-D. villosum#4 alien chromosome lines
Source: BMC Genomics. 2019 Apr 15;20:289. doi: 10.1186/s12864-019-5630-4 (PMC6466811; doi:10.1186/s12864-019-5630-4)
Supplement: Supplementary file 2 — Localization of the 76 developed PCR markers in wheat, barley, and D. villosum#4, and their primer sequences and amplified sizes. 1: Chromosomal localization of the corresponding homologous sequences of the developed markers in wheat; 2: Chromosomal localization of the corresponding homologous sequences of the developed markers in barley; 3: Chromosomal localization of the corresponding sequences of the developed markers in D. villosum#4. (DOCX 24 kb) [file 12864_2019_5630_MOESM2_ESM.docx]

**Additional file 2:** Localization of the 76 developed PCR markers in wheat, barley, and *D*. *villosum#4*, and their primer sequences and amplified sizes

| Molecular markers | Localization in wheat^1^ | Localization in barley^2^ | Localization in *D*. *villosum#4*^3^ | Forward primer sequence | Reverse primer sequence | Product size |
| --- | --- | --- | --- | --- | --- | --- |
| 1V-1 | 1AS | 1H | 1V | TTCCTTCACTCTCTTCTCTTGCTTGC | AATACTTCCTGGATTCCTCGTCCGA | 389 |
| 2V-1 | 5DS | 5H | 2V | CGACAGCCTCTCCATCTTCTCCT | CAAGCCCTTCTCAGCCTCCAATG | 364 |
| 2V-2 | 7DL | 7H | 2V | CACCACTATCTTCCTGCTCAGTTGTTG | GGCTACCACACCAAGTCCTCCAA | 424 |
| 2V-3 | 2DS | 2H | 2V | AGACGGAGCATGTGAGTA | CCTGAGTGAGTGATTACGAT | 258 |
| 2V-4 | 2DS | 2H | 2V | AGACGGAGCATGTGAGTA | CTGAGTGAGTGATTACGATTG | 257 |
| 2V-5 | 2DS | 2H | 2V | TGCGTATGAGATATGCTACT | GTCTGAACCATAGGCTGAG | 381 |
| 2V-6 | 2BS | 2H | 2V | CAGGTAACACTTAGCAACTTC | CCACAGTCTGAACCATAGG | 280 |
| 2V-7 | 2DS | 2H | 2V | TGCGTATGAGATATGCTACT | CATCCACAGTCTGAACCAT | 389 |
| 2V-8 | 2DS | 2H | 2V | CATAGAATGAGCGAGAAGTTG | CGAAGTTGCTAAGTGTTACC | 476 |
| 2V-9 | 2BS | 2H | 2V | CTCCTGGCGATAAGGTTC | TGCCTCTGCTTAAGATGTT | 161 |
| 3V-1 | 5BL | 4H | 3V | TCCATCATAGCACCTTCAGACTCAAG | GACAACTCGGCAATCACCAAGGA | 377 |
| 3V-2 | 5DL | 3H | 3V | GGCAACTCAAATTATAGGATCACGAC | GCAAGGCGGAGTAGCTCACA | 243 |
| 3V-3 | 5DL | 3H | 3V | TTCGTCATCTTTGTTGACATGGCAA | GCAAGGCGGAGTAGCTCACA | 263 |
| 3V-4 | 5DL | 3H | 3V | GGCAACTCAAATTATAGGATCACGAC | ACTATATGTTGATGACGAGGAGCAA | 332 |
| 3V-5 | 3AL | 3H | 3V | TTAGAGCGACGACAACTATGC | CAGTATAAGATAGGAGAAGCGACAG | 175 |
| 3V-6 | 3AL | 3H | 3V | CCTCCCGGCTAAACAAGCA | GCAAGATAAATACGCACTCCAAAG | 141 |
| 3V-7 | 3DL | 3H | 3V | GTTATATCGGTTGAGGCGTCTATAC | AACAGTGAGTTCTTCAGGACAGA | 341 |
| 3V-8 | 3AL | 3H | 3V | GGAGGCTGTAATACTGGATTCTG | TGGCTCTGATGCTGATGCT | 559 |
| 3V-9 | 3AL | 3H | 3V | GGAGGCTGTAATACTGGATTCTG | GCTCTGATGCTGATGCTGTTA | 557 |
| 3V-10 | 3AL | 3H | 3V | GGAGGCTGTAATACTGGATTCTG | GATACTGGCTCTGATGCTGATG | 564 |
| 3V-11 | 3DL | 3H | 3V | CTCGTCGGTCTCAGAAGTCAA | TCCACAGAATCATCGGCTCTC | 760 |
| 3V-12 | 3DL | 3H | 3V | CCTCCTCTTCCTCCTCTTCC | TCGCACCATCACCGTACTT | 499 |
| 3V-13 | 3DL | 3H | 3V | TGGTTCTGATACTGGTGTAGCA | ACCGCCATAGTAGCATACCTTAT | 1560 |
| 3V-14 | 3DL | 3H | 3V | GGACGGATGTAGTCTTGTTCAA | CTCGTATCGTACTGCTACTCA | 593 |
| 3V-15 | 3DL | 3H | 3V | CTTCGCTCACATGACCGTATT | CTTCGCTCACATGACCGTATT | 258 |
| 3V-16 | 3DL | 3H | 3V | GTCCACCAAATCACATCAAACA | GCTCTCACAAGTCACAACAATT | 180 |
| 3V-17 | 3DL | 3H | 3V | ACCATATACTTCGGTGGAACATAC | GCATAGTTACTCTATCACAGACTCA | 304 |
| 3V-18 | 3DL | 3H | 3V | TACCATATACTTCGGTGGAACATAC | GCATAGTTACTCTATCACAGACTCA | 305 |
| 3V-19 | 3DL | 3H | 3V | TGCTCTTCACAGTTCATCTCCT | AGACAAGTTCAGTTCCACACTC | 364 |
| 3V-20 | 3AS | 3H | 3V | TGGTTGCTTCTCAGTTGTGTTG | TACTCGGATAGTGCCTTGTTGA | 237 |
| 3V-21 | 3AS | 3H | 3V | GTTGCTTCTCAGTTGTGTTGGA | TACTCGGATAGTGCCTTGTTGA | 235 |
| 3V-22 | 3AS | 3H | 3V | TGGTTGCTTCTCAGTTGTGTTG | CGGATAGTGCCTTGTTGATGAC | 233 |
| 3V-23 | 3AS | 3H | 3V | CAACATTACGGTGGCGGATCA | GATGTCTTCATGTGGCACAGGAA | 255 |
| 3V-24 | 3DS | 3H | 3V | GAGAACTGCTCAACATGACAATAAG | CAACAGTATCATCAATGGAGGTCTT | 144 |
| 3V-25 | 3AS | 3H | 3V | CGATTAGTCCATCCTCTCCATTC | GGTCTTCGACGACGACATCT | 186 |
| 3V-26 | 3DS | 3H | 3V | TCGCCAGCACCAACCAAT | CAGCACAGCACACCAATGAA | 686 |
| 3V-27 | 3AS | 3H | 3V | GTGACACCAATAGAAGGCAGAA | GGAGGAGCATACCGTGGAA | 403 |
| 4V-1 | 7DS | 7H | 4V | AAGAACATGGACCAGATACGCAACA | TGGCACCAGCATTGTCGAACTC | 1100 |
| 4V-2 | 7AS | 7H | 4V | GCAGCAGGCAGCACATCATACA | TTGGAGTAGCGACGACGAGGAT | 280 |
| 4V-3 | 5DL | 5H | 4V | GAGGTCGTTCTCTGAGGTCATCGT | GCTCCTTGGAATTGGCGGCTTC | 510 |
| 4V-4 | 7DS | 4H | 4V | AGCACCGACGACGACGAAGA | ACTGACGCACGCATGGCATC | 300 |
| 4V-5 | 4AL | 4H | 4V | CGTAAGGTACACGCTGTTAGC | TCCCGATTTGTAACATTAG | 340 |
| 4V-6 | 4DL | 4H | 4V | CCGACCTTTCCCTTGTTTATA | TAGCCTTGGTGAAAGCAAAC | 192 |
| 4V-7 | 4BL | 4H | 4V | CGGTGTAATAATTGCCTTCAACTG | GCGTGCTAATCTTGCTATCTTCT | 354 |
| 4V-8 | 4DL | 4H | 4V | ACACCGTCGCCATACTCAA | GTGCTAATCTTGCTATCTTCTGAAC | 789 |
| 4V-9 | 4BL | 3H | 4V | TGGCGAACATCTTGCTCATC | AAGGAGGCTGATACTGCTGAA | 245 |
| 4V-10 | 4DL | 6H | 4V | TGGTTATGAAGGAACTCGTGGTA | AGGCTGATACTGCTGAAGACTT | 1200 |
| 5V-1 | 5BL | 5H | 5V | GCGAAATCATGCGACTCCAATAACTAA | CGGCGTCCTCATCAACAACCT | 547 |
| 5V-2 | 5AL | 5H | 5V | GGTTCACTTGTTGCTCTGTTACATCTTC | CCTCACATTGGCTATGCTGGACTATAC | 286 |
| 5V-3 | 5BL | 5H | 5V | GGCTGATCCGACGATGAAGAAACC | TGTCCTGATAACCTCCACCACTCC | 373 |
| 5V-4 | 5DL | 5H | 5V | TCGCAGTATGTACCACCACCAAT | GGCCACATAATGTACCTACACTTAGAT | 376 |
| 5V-5 | 5DL | 5H | 5V | GTCGCAGTATGTACCACCACCAA | ACAACCAGATAGTCAAGCTAACCTACA | 234 |
| 5V-6 | 5BL | 5H | 5V | CGAATCAAGAACGAACTCAGCAGAC | CCTCTCCTCTCCTCTCCTCATCTC | 208 |
| 5V-7 | 5DL | 5H | 5V | CGAATCAAGAACGAACTCAGCAGAC | CCTCTCCTCTCCTCTCCTCATCTC | 208 |
| 5V-8 | 5DS | 5H | 5V | CACCATGCAGTAAACACCACCTCA | TCATTGATAGGCTGAACACCAAGGAA | 260 |
| 5V-9 | 5DL | 5H | 5V | GGAGGAGGTCAACGAGAACGAGAT | TACTATACAGCACAGCCGCAGACTAT | 469 |
| 5V-10 | 5DL | 5H | 5V | GAAGCACATGCAGTAGCGGTAGG | TTAGAAGCCGAGTCCACTCCAGTC | 202 |
| 5V-11 | 5BL | 5H | 5V | AAGCCAGAACAACTCACAACACCTT | CTCCAAGCACTTCCTGTCACCATAC | 421 |
| 5V-12 | 5BL | 6H | 5V | CTCGTGTCAGTGACCAGCTTGTG | AACCAATGGATCACCTCGTCATCAAC | 244 |
| 5V-13 | 5BL | 5H | 5V | TCCGCCCAGATGCCGAGAAT | AGACCGTTGTCCACATGCTCAC | 472 |
| 5V-14 | 5BL | 5H | 5V | GGTCGTCTCCTGGTTCCAGAAGA | AGTTCGGCGTCCTCATCAACAAC | 284 |
| 5V-15 | 5DS | 5H | 5V | GATAGAGGTTTCACAGGTTGTCTTCAC | AGAGGTAGGGATCAAAGTGGCAAAG | 239 |
| 6V-1 | 6DL | 6H | 6V | CCGTGCGACAGAACAGAAGTGA | GCAATCAGCCACATACAGGTCATC | 1500 |
| 6V-2 | 6DL | 6H | 6V | AACCAACCACCACTCCAATCTCC | CCTTGCCATCAATGTCATACACCTT | 480 |
| 6V-3 | 6DL | 6H | 6V | AGCAGACGAGGACGCAACAAG | GCAATCAGCCACATACAGGTCATC | 480 |
| 6V-4 | 5BL | 1H | 6V | ACCATCCGTCTTGGCATATTCAGTC | AGCAGTGAGCAGTTGTCTTCTTGTT | 732 |
| 6V-5 | 6BS | 6H | 6V | GCCAGTAAGATTCCGTATGCCCTCT | ACCTTCCTCACAACACTCCTTCTCT | 891 |
| 6V-6 | 6BS | 6H | 6V | ACTGCCCAAATGCTAGAGATGTTCA | CTCAGTCCACGACAGACACTCAAG | 218 |
| 6V-7 | 6DS | 2H | 6V | CGCAAGACCTCAACGCCAACT | AGGAGGCAGTATAGCACGACCAATA | 540 |
| 6V-8 | 6BS | 6H | 6V | TTGGGACGGAGGGAGTACATACG | ACCAGCAGGCACAACGAGGA | 600 |
| 6V-9 | 6AS | 4H | 6V | GGTTGTTATCTGTCTGTCGGTGGAA | CGTCAGCAGCAGTTGTATCATGTTC | 698 |
| 6V-10 | 7BS | 7H | 6V | AACGAAGATACCAAGGCGATGCT | AGCGATCACCTCCACCGTCA | 471 |
| 6V-11 | 7DS | 7H | 6V | AGGACAAGAAGCCTCGCTAGAAGAT | CGGTCATGCATCGGCCAACA | 308 |
| 6V-12 | 7BS | 7H | 6V | AGGTGCGTGCCAAGGAGGAA | CTTCGCCAGCATCACCAAGGT | 312 |
| 6V-13 | 2DL | 4H | 6V | GCAACGCCATCAAGACCAGTGTAT | TGACTCAACCTATTACGCAGGACGAT | 839 |
| 7V-1 | 5BL | 6H | 7V | TACGAGATGGAGCGTGACGAAGG | CGATGTTGTCATCCCGGACAGG | 234 |

1: Chromosomal localization of the corresponding homologous sequences of the developed markers in wheat.

2: Chromosomal localization of the corresponding homologous sequences of the developed markers in barley.

3: Chromosomal localization of the sequences of the developed markers in *D*. *villosum#4*.
